# Transactions of the N. York State Medical Society

**Published:** 1844-06

**Authors:** 


Art. VI.—*Transactions of the New York State Medical Society. Vol. VI. Part. I.* Albany: Joel Munsell. 1844.

This is a collection of interesting papers. The second one, by Dr. Blatchford, discusses a curious question connected with equivocal generation. Dr. B. was called on to testify in a suit of slander, in which one woman was accused by another of having become pregnant by a dog. He declares the impossibility of such an occurrence, and enters into many striking details to prove his position. In order to procreation, he says, there must be an agreement in the three following particulars:

"1. Both species must possess organs of generation exactly alike.

2. The time they go with young must be the same.

3. They must agree in their manner of copulation.

Hybrids among animals occur only in a very few classes. There is that of the horse and the ass kind, but here we find the female ass and the mare furnished with organs of generation precisely similar, their period of gestation is the same, being a little over eleven months, and so is their manner of copulating; thus there is a concurrence of these three important particulars, essential to pregnancy in cross breeds. The same similarity occurs between the male organs of generation in the jack and the stud as occurs between those of the females of the same species. But here a circumstance should be mentioned which shows very strongly the antiphysical nature of such abnormal productions. The hybrids of these animals never possess the power of reproduction; although as far as the investigations of the anatomist can ascertain, they are furnished with all the necessary organs of generation, and the male mule is vastly more lecherous than either the jack or the stud.

"Another instance is that between the sheep and the goat. Here, likewise, we find the three essentials present; the organs of generation, and the manner of copulation are the same, and they both go with with young five months."

Experiments have been made again and again, he says, upon the dog, wolf, and fox, to see whether they would cohabit promiscuously. He adds:

"The organs of generation are alike in all three, and the manner of copulation is the same likewise; but a difference exists between them in the period they go with young. The fox goes six weeks, the dog nine, and the wolf fourteen. This is doubtless the great barrier, which, together with the almost irreconcilable hatred subsisting among them toward each other, the wise Creator has set up to prevent their promiscuous union. If so, we may safely predict that every succeeding experiment will be alike unsuccessful."

The popular belief is opposed to this. It is generally supposed that intercourse between the dog and the wolf is fruitful. Buffon, however, maintains the contrary, and he investigated the point with great labor—procured pups of the two races, brought them up together, and kept them in retired places; but in three years they were never observed to have sexual intercourse. If animals agreeing in almost every particular do not possess this power, it cannot be expected, says Dr. B., in those who differ so widely as man and dog. Having stated the difference in their organs of generation, he adds:

"The two species differ as widely in the construction of the uterus, and these differences, to say nothing of one being uniparous, or bearing one at a birth, and the other multiparous, or bearing many at a birth, are sufficient to make a great difference in the manner of copulation. In our own species it is a muscular propulsion of the sperm by the male, almost entirely unassisted, into the uterus of the female. In the dog, on the contrary, it is a kind of muscular secretion, or mechanical compression, produced entirely by the contraction of the female organs, excited to their highest degree by the presence of the male. In the one, the male is the active agent in making the deposite, while the female remains nearly passive; in the other the male remains almost entirely passive while the female is the active agent.

"If therefore it be true, as I think I have made it abundantly appear, that a certain essential power be wanting in both individuals, a power without which impregnation is impossible, even admitting all other things to favor the opposite result, does it not approach very nearly to a demonstration, that the object contemplated can never be effected?

"But if these differences of structure are not sufficient to prove my position, there is the very wide difference between the two individuals in the period of gestation, while in one it is forty weeks, in the other it is only nine."

Any one curious about such matters will find much more to the same point in this paper. The volume is one which does credit to the Society from which it emanates.

